# Supplementary figures and images for: From patient to tumor organoid: Culture protocol choice controls glioblastoma tumor architecture and identity
Source: Brain Pathol. 2026 Jul 26:e70125. Online ahead of print. doi: 10.1111/bpa.70125 (PMC13402234; doi:10.1111/bpa.70125)

(A)

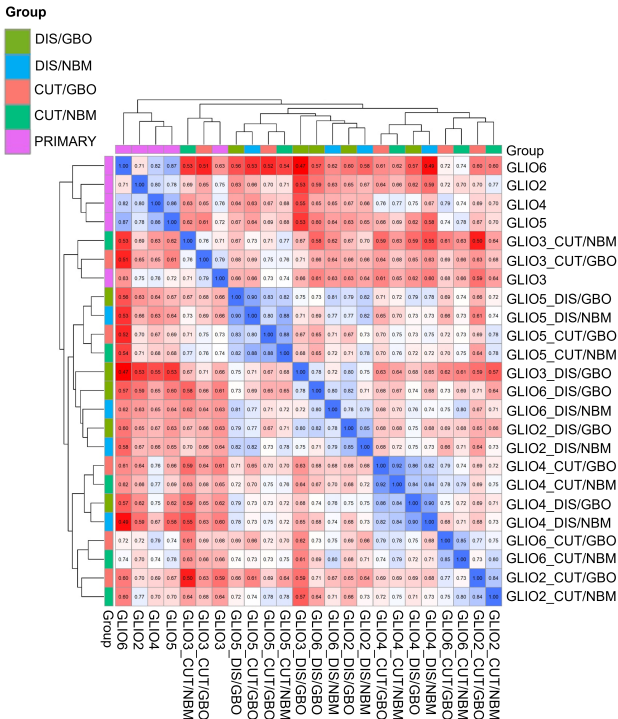

(B)

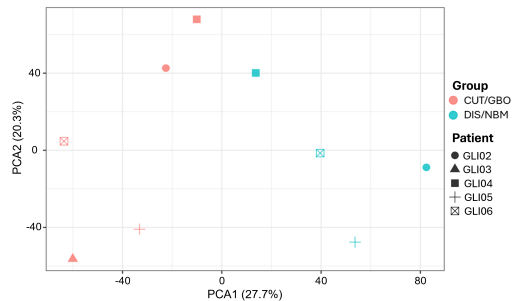

Supplement: Supplementary file 1 — Figure S1. Assessment of biological variability and culture medium effects across all culture conditions. (A) Pearson's correlation analysis of proteomic profiles across all biological replicate samples, illustrating relationships between culture conditions and primary tumors. (B) PCA of CUT/GBO and DIS/NBM organoids across biological replicates. [file BPA-9999-e70125-s002.pdf]
