# Supplementary material for: From patient to tumor organoid: Culture protocol choice controls glioblastoma tumor architecture and identity
Source: Brain Pathol. 2026 Jul 26:e70125. Online ahead of print. doi: 10.1111/bpa.70125 (PMC13402234; doi:10.1111/bpa.70125)
Supplement: Supplementary file 2 — Table S1. Clinical and pathological data of the patient samples used. [file BPA-9999-e70125-s005.docx]

**Supplementary Table 1:** Clinical and pathological data of the patient samples used

| Code | Clinical data | | | | | Pathology | | | | | | |
| --- | --- | --- | --- | --- | --- | --- | --- | --- | --- | --- | --- | --- |
|  | **Gender** | **Age at the surgery** | **Localization** | **SVZ involvement** | **Epilepsy** | **Final integrated diagnosis according to the WHO CNS5** | **IDH1 R132H IHC** | **IDH1/2 sequencing** | **pMGMT methylation** | **p53 IHC** | **ATRX IHC** | **Ki-67** |
| GLIO1 | Female | 54 | Left frontal lobe | NO | YES | Glioblastom IDH-wildtype, WHO G4 | wildtype | wildtype | unmethylated | mutant | retained | 30% |
| GLIO2 | Male | 51 | Left frontal lobe | YES | NO | Glioblastom IDH-wildtype, WHO G4 | wildtype | wildtype | unmethylated | wildtype | retained | 10% |
| GLIO3 | Male | 57 | Left frontal lobe | YES | NO | Glioblastom IDH-wildtype, WHO G4 | wildtype | wildtype | unmethylated | mutant | retained | 25% |
| GLIO4 | Male | 73 | Left temporo-parietal lobes | NO | NO | Glioblastom IDH-wildtype, WHO G4 | wildtype | wildtype | methylated | mutant | retained | 30% |
| GLIO5 | Male | 75 | Left temporal lobe | NO | NO | Glioblastom IDH-wildtype, WHO G4 | wildtype | wildtype | unmethylated | mutant | retained | 40% |
| GLIO6 | Female | 64 | Right temporo-parietal lobes | YES | NO | Glioblastom IDH-wildtype, WHO G4 | wildtype | wildtype | unmethylated | mutant | retained | 20% |
