# Supplementary material for: From patient to tumor organoid: Culture protocol choice controls glioblastoma tumor architecture and identity
Source: Brain Pathol. 2026 Jul 26:e70125. Online ahead of print. doi: 10.1111/bpa.70125 (PMC13402234; doi:10.1111/bpa.70125)
Supplement: Supplementary file 3 — Table S2. List of antibodies used for IHC. [file BPA-9999-e70125-s004.docx]

**Supplementary Table 2:** List of antibodies used for IHC

| Antigen | Catalogue number | Host/  antibody type | Clone | Working dilution | Manufacturer | Location |
| --- | --- | --- | --- | --- | --- | --- |
| GFAP (glial fibrillary acidic protein) | M0761 | Mouse monoclonal | 6F2 | 1:200 | Dako Agilent | Santa Clara, CA, USA |
| S100β | Z0311 | Rabbit polyclonal | Polyclonal | 1:1000 | Dako Agilent | Santa Clara, CA, USA |
| NSE (neuron-specific enolase) | 06648568001 | Mouse monoclonal | MRQ-55 | Ready-to-use (RTU) | Cell Marque | Rocklin, CA, USA |
| CD31 | CI948C01 | Mouse monoclonal | JC70A | 3:100 | Innovative Diagnostik-Systeme GmbH & Co. KG | Hamburg, Germany |
